# Crosstalk between gut microbiotas and fatty acid metabolism in colorectal cancer

**DOI:** 10.1038/s41420-025-02364-5

**Published:** 2025-02-26

**Authors:** Hao Zhang, Yuan Tian, Chunjie Xu, Miaomiao Chen, Zeyu Xiang, Lei Gu, Hanbing Xue, Qing Xu

**Affiliations:** 1 Department of Gastrointestinal Surgery, Renji Hospital, School of Medicine, Shanghai Jiao Tong University, 160 Pujian Road, Shanghai, 200127 China; 2 Department of Radiology, Huashan Hospital, National Center for Neurological Disorders, State Key Laboratory of Medical Neurobiology, Fudan University, Shanghai, 200040 PR China; 3 Division of Gastroenterology and Hepatology, Key Laboratory of Gastroenterology and Hepatology, Ministry of Health, Renji Hospital, School of Medicine, Shanghai Jiao Tong University, Shanghai, China

**Keywords:** Cancer microenvironment, Cancer metabolism, Rectal cancer

## Abstract

Colorectal cancer (CRC) is the third most common malignancy globally and the second leading cause of cancer-related mortality. Its development is a multifactorial and multistage process influenced by a dynamic interplay between gut microbiota, environmental factors, and fatty acid metabolism. Dysbiosis of intestinal microbiota and abnormalities in microbiota-associated metabolites have been implicated in colorectal carcinogenesis, highlighting the pivotal role of microbial and metabolic interactions. Fatty acid metabolism serves as a critical nexus linking dietary patterns with gut microbial activity, significantly impacting intestinal health. In CRC patients, reduced levels of short-chain fatty acids (SCFAs) and SCFA-producing bacteria have been consistently observed. Supplementation with SCFA-producing probiotics has demonstrated tumor-suppressive effects, while therapeutic strategies aimed at modulating SCFA levels have shown potential in enhancing the efficacy of radiation therapy and immunotherapy in both preclinical and clinical settings. This review explores the intricate relationship between gut microbiota, fatty acid metabolism, and CRC, offering insights into the underlying mechanisms and their potential translational applications. Understanding this interplay could pave the way for novel diagnostic, therapeutic, and preventive strategies in the management of CRC.

## Facts

1. Metabolism of colorectal cells shapes the microbiome. Metabolites are the primary mediators of information exchange between the gut and the microbial community.
2. Fatty acid metabolism plays an integral role in the development of colorectal cancer cells.
3. It is well known that different types of fatty acids have different roles, so we further explored the link between the metabolism of different fatty acids and the gut microbes during the development of colorectal cancer.

## Open questions

1. What happens to both fatty acid metabolism and gut microbes in colorectal cancer patients?
2. What exactly is the link between fatty acid metabolism and gut microbes?

## Introduction

Colorectal cancer (CRC) is a large and expanding global health issue, with a more serious epidemiological profile and an earlier age tendency [1]. The relationship between gut microbiotas and CRC has attracted much scientific interest in recent years [2]. Emerging epidemiological evidence suggests notable shifts in both microbiota composition and metabolism in CRC patients, underscoring the complex interactions between gut microbes and the disease [3, 4]. It also exhibits that the gut microbiotas influence the adaptive and innate immune systems, which regulate the pathogenesis of inflammatory bowel disease and the development of CRC [5]. Gut microbiotas play a crucial role in producing diverse metabolites that contribute to maintaining host health [6]. They adjust to alterations in the gut ecology by modifying substances produced by bacteria, such as decreasing SCFA production through microbial fermentation. Additionally, cancer cells in tumor microenvironments, where nutrient availability shifts throughout tumor progression, utilize lipid metabolism to sustain rapid proliferation, survival, migration, invasion, and metastasis [7]. Metabolites generated from gut microbiota, particularly SCFAs, serve various functions in host balance. SCFAs, which are the primary source of energy for colon cells, influence systematic immune system reactions and function as tumor suppressors by altering the expression of genes through epigenetic mechanisms [8]. Although the prognosis for CRC has steadily improved in recent decades, there is still a need for better therapy strategies. As a result, a greater awareness of CRC-associated microbiota metabolism and fatty acid metabolism holds the potential to enhance the accuracy of diagnosis and lead to CRC treatment methods. This review aims to explore the complex crosstalk between gut microbiota and fatty acid metabolism in CRC, providing insights that may inform future diagnostic and therapeutic advancements in CRC management.

### Remodeling of fatty acid metabolism in CRC

#### Effects of fat acid over-absorption on the CRC

High-fat consumption and obesity rates are rising worldwide, with both conditions being associated with the development of aggressive cancers [9]. It is well known that CRC is more regarded as a disease of affluence, and it has a lot to do with people’s unhealthy diets. What we can see is that the countries with a higher prevalence of CRC globally are mainly concentrated in the Western developed countries [10, 11]. It is worth noting that more than 33 percent of American adults are overweight or obese due to a high fat intake, a population that is rapidly increasing in Asia as the economy rises [12], and which must have a lot to do with the Western diet as we think of it. Consistent with this, the incidence of malignant tumors is increasing progressively, with High-fat diets (HFDs) and obesity now recognized as key risk factors for several malignant tumors, including breast, colorectal, and liver cancers [13–15]. For instance, obesity associated with dietary fat is associated with the development of breast cancer in postmenopausal women [16]. Moreover, studies of the dietary composition of populations have shown that those who consume more meat and animal fat have a significantly higher incidence of colorectal cancer [17]. Animal fats, especially those that have a higher Omega-6/ Omega-3 fatty acid ratio, are more strongly associated with the development of CRC [18, 19].

HFDs, the main patterns include the Western diet, which is based on saturated fatty acids, the Mediterranean diet, which is based on Omega-3 polyunsaturated fatty acids, and the Asian regional diet, which is based on Omega-6 polyunsaturated fatty acids [20]. Especially the Western diet, which is characterized by fat-rich, red, and processed meat, is now recognized as one of the risk factors for CRC [21]. Saturated Fatty Acids (SFAs) are thought to be closely related to colorectal cancer development, and they can contribute to the development of CRC through a variety of pathways [22]. For example, SFAs can induce the secretion of pro-inflammatory factors such as TNF-α, IL-6, and IL-1β through activation of the Toll-like Receptor 4 (TLR4) and Nuclear Factor κB (NF-κB) signaling pathways, leading to the formation of a chronic inflammatory environment [23, 24]. It can also increase the production of Reactive Oxygen Species (ROS), leading to elevated levels of oxidative stress. Accumulation of ROS can cause DNA damage and mutations, thereby promoting the development of cellular cancer [25]. Similarly, excessive intake of SFAs can disrupt the intestinal barrier function, leading to intestinal flora dysbiosis and promoting the overgrowth of harmful bacteria, such as Acanthamoeba [26]. In contrast, Omega-3 fatty acids have been reported to have anti-inflammatory effects by inhibiting the expression of interleukin-1 β (IL-1 β), tumor necrosis factor-alpha (TNF-α) and IL-6, and this is one of the reasons why the Mediterranean diet is considered to be a healthy diet [27]. However, metabolites of Omega-6 fatty acids, such as prostaglandins, thromboxanes, and leukotrienes, promote thrombosis, vasoconstriction, and atherosclerosis, and reduce the concentration of Omega-3 fatty acid metabolites [19, 28]. Several studies focusing on diet-gene interactions have reported that dietary Omega-6 fatty acids promote atherosclerosis, while Omega-3 fatty acids inhibit leukotriene-mediated inflammation [29]. Thus, Omega-6 fatty acids have a stronger pro-inflammatory effect relative to Omega-3 fatty acids [30]. However, this does not allow us to simply classify Omega-6 as strictly pro-inflammatory, and we are currently more concerned with the effects of the Omega-6/Omega-3 ratio on human health. It has been reported that the ratio of Omega-6/Omega-3 fatty acids in the human diet has increased from approximately 1 to 20 over the past 30 million years [31], and the ratio of dietary Omega-6/Omega-3 fatty acids can influence the ratio of Omega-6 to Omega-3 fatty acids in the body [32]. The current United Nations recommendation for the Omega-6/Omega-3 ratio is 5:1, so a significant imbalance in the ratio between the two may be a more critical factor in the development of inflammation, or even tumors, than the effect of a single unsaturated fatty acid changing in the body [33].

Further studies also confirmed that fats may be cancer-causing through the production of oxides and fatty acids, and different components of lipids have various effects on CRC [34]. Dietary cholesterol intake is positively associated with the risk of developing CRC [35]. The quantity of circulating fat in the body rises rapidly with the consumption of high-fat foods, which suppresses immune surveillance and favors tumor progression [36]. For instance, HFDS-induced obesity reduces the production of granzyme and cytokines (TNF-γ, TNF-α), which in turn causes the depletion of CD8+ tumor-infiltrating lymphocytes (TILs) and ultimately speeds up tumor growth in a mouse model of colorectal cancer [37–39]. Similarly, the ubiquitous lipid ligand-activated transcription factor peroxisome proliferator-activated receptor (PPAR) directly influences adipocytes to enhance fatty acid oxidation when activated by dietary factors such as fatty acids [40–42]. Persistent consumption of HFDS alters the expression of genes involved in inflammation, angiogenesis, and cell proliferation, which in turn promotes CRC [43]. This may enhance their ability to initiate tumors while regulating cell proliferation differentiation apoptosis [44]. In addition to creating an immunological environment conducive to tumor growth, HFDS-induced lipid accumulation is particularly important for tumor metastasis as well as recurrence [45]. Once the tumor cells spread or become tolerant to treatment, they accelerate lipid intake. The excess lipid accumulation caused by HFDs promotes the transfer of migrating tumor cells through the bloodstream to other organs [46, 47]. Lipid testing of CRC patients revealed that TC, LDL-C, and LDL-C/HDL-C ratios were significantly higher in patients with distant metastases than in patients without metastases [48]. The LDL-C and LDL-C/HDL-C ratios are effective biomarkers, and their elevation indicates that patients with CRC are more likely to develop lymph node metastasis [49]. Overall, for CRC, HFDS can enhance cancer cell metastasis by increasing lipid availability and altering the lipid balance throughout the body and within the tumor, thereby reducing immune surveillance.

#### Alterations of fatty acid metabolism in CRC

Recent studies have shown that energy metabolism plays an important role in tumor development [50–52]. In addition to alterations in glucose and amino acid metabolism, alterations in lipid metabolism are an important feature of tumor metabolism [53]. With the continuous intake of high-fat diets, fatty acid metabolism patterns also undergo significant changes in intestinal cells. Fatty acids are an important source of energy for intestinal cells, and they play an important role in cell membrane maintenance, signaling, and inflammatory regulation [54]. They are essential synthetic components of phospholipids, which are important structural molecules of cell membranes. Therefore, abnormal lipid metabolism can lead to molecular changes such as altered cell membrane structure [55], abnormal cell signaling, imbalance in energy homeostasis, and disturbances in gene expression and protein distribution, which in turn affects a series of cellular functions such as cell proliferation, differentiation, metabolism, apoptosis, and information transfer [56, 57].

For normal and CRC intestinal cells, they share some similarities and differences in fatty acid metabolism. Both of them can catabolize fatty acids by fatty acid β-oxidation (FAO) to produce ATP and the fatty acid metabolism of both is closely related to the composition and metabolic activities of the intestinal microbiotas like short-chain fatty acids, such as butyric acid, which have a regulatory effect on both normal and cancer intestinal cells [58]. However, for CRC enterocytes, they have significant differences in the intensity of fatty acid metabolism, and these differences are closely related to the energy requirements, biosynthesis, and signaling needs of cancer cells [53]. Firstly, in terms of fatty acid synthesis, normal enterocytes take up fatty acids mainly through diet and do not significantly activate de novo fatty acid synthesis. In contrast, the biosynthesis of CRC tumor cells is dependent on continuous de novo fatty acid synthesis [59]. The de novo synthesis is regulated by fatty acid synthase (FASN) via acetyl-CoA and malonyl-CoA [58]. It is well known that three hallmarks of metabolic dysregulation in cancer cells are shared: enhanced glycolysis and glutaminolysis, and elevated de novo lipogenesis [60]. Thus, FASN overexpression occurs frequently in CRC enterocytes. Secondly, in the context of fatty acid catabolism utilization, FAO is a critical component of cellular metabolism, playing a vital role in supporting cancer cell survival by providing the necessary energy [61]. FAO comprises a series of cyclic processes that progressively shorten fatty acid chains to generate NADH, FADH₂, and acetyl-CoA [62]. Both of the former are linked to the mitochondrial electron transport chain complex (ETC) to generate ATP, while the latter enters the Krebs cycle to generate citrate, which is then transferred to the cytoplasm for NADPH generation [63]. During this process, carnitine acts as a carrier for acyl-CoA, facilitating the transport of fatty acids into the mitochondrial matrix and supporting the further processing of long-chain fatty acids within the mitochondria [64]. Long-chain acyl-CoA synthetases (ACSLs) are key enzymes responsible for converting free long-chain fatty acids into fatty acyl-CoA esters [65]. Among them, ACSL3 plays a pivotal role in tumor progression by promoting fatty acid uptake and β-oxidation [66]. In CRC cells, epithelial-mesenchymal transition (EMT) induced by TGF-β1 significantly enhances fatty acid uptake and β-oxidation. ACSL3 mediates TGF-β1-induced metabolic reprogramming of fatty acids, thereby facilitating EMT and metastasis in CRC cells [67, 68]. These findings highlight a mechanistic link between TGF-β1-induced energy metabolism remodeling and invasive phenotypes, suggesting that ACSL3 may promote cancer cell migration and metastasis by stimulating FAO [69–71]. Therefore, studies targeting ACSL3 and FAO in terms of changes in fatty acid metabolism may have great therapeutic potential in the development and metastasis of CRC.

**Fig. 1:**
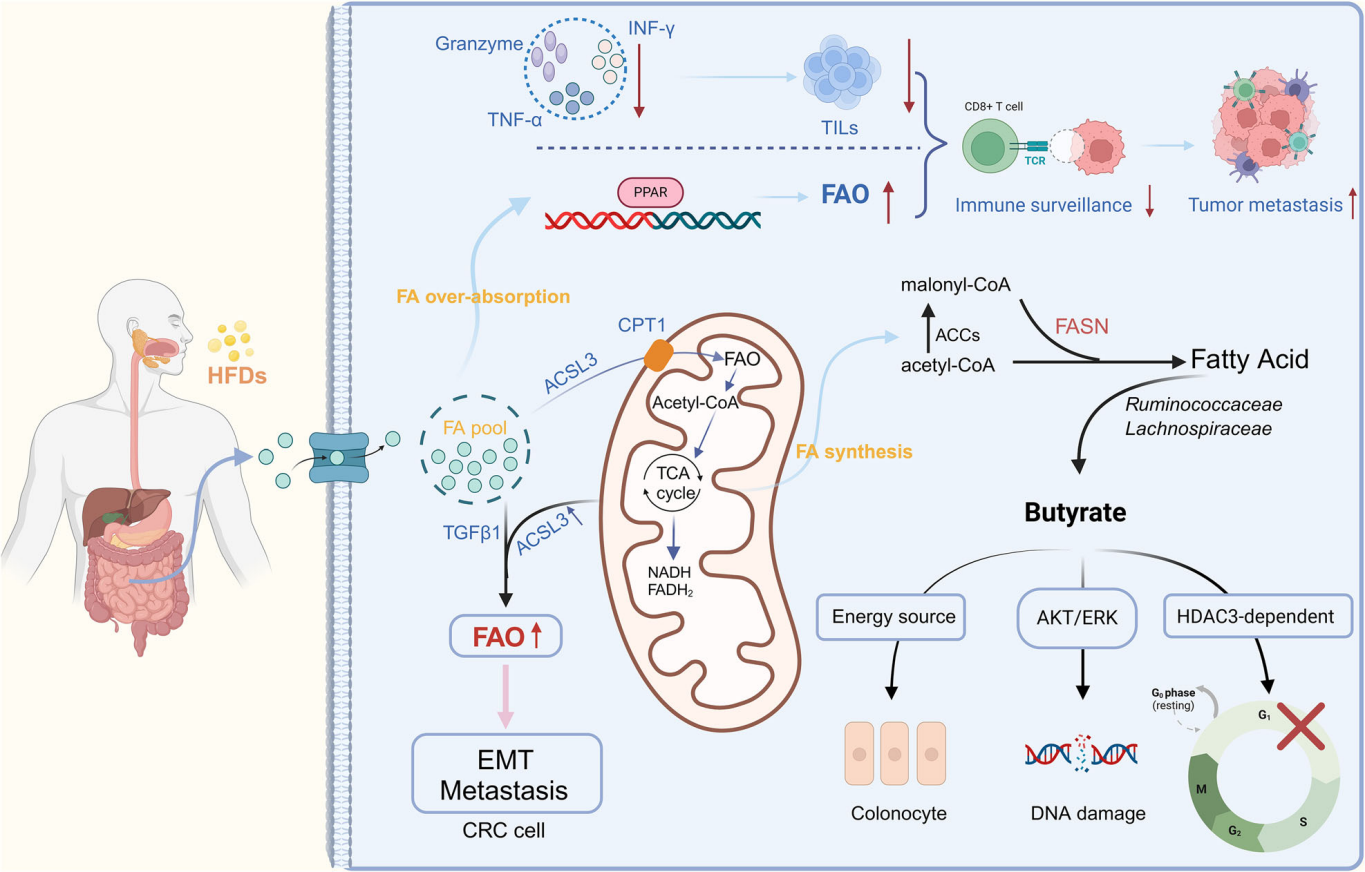
Alterations of fatty acid metabolism in CRC. FASN overexpression occurs frequently in CRC enterocytes. FAO comprises a series of cyclic processes that progressively shorten fatty acid chains to generate NADH, FADH₂, and acetyl-CoA.

In addition to differences in synthesis and degradation, normal intestinal cells and CRC cells exhibit significant disparities in fatty acid metabolism regulatory pathways [72]. The metabolism of normal intestinal cells is tightly regulated by energy sensors such as AMP-activated protein kinase (AMPK) [73]. In contrast, CRC cells often exhibit suppressed or reprogrammed AMPK activity, coupled with activation of the mTOR signaling pathway, to support the rapid proliferation of cancer cells [74]. Meanwhile, normal intestinal cells rely minimally on lipid storage, utilizing fat reserves only under specific conditions such as starvation. CRC cells show enhanced lipid droplet formation, serving as an energy reservoir to support their rapid growth and increased antioxidant demands [75]. Furthermore, gut microbiotas play a significant role in driving differences between normal intestinal cells and CRC cells [76]. In normal intestinal cells, gut microbiotas contribute to the production of SCFAs such as butyrate and acetate, which provide energy to the host, promote anti-inflammatory effects, and maintain intestinal barrier integrity [77]. In contrast, CRC cells are associated with gut microbiota imbalances, such as an increase in harmful bacteria and a decrease in beneficial microbes, which may result in disrupted fatty acid metabolism [78]. For example, the proliferation of harmful bacteria, including sulfate-reducing bacteria and pro-inflammatory microbial populations, produces metabolic byproducts such as hydrogen sulfide (H₂S) [79]. These byproducts can damage the intestinal barrier, facilitating tumor initiation and progression. (Fig. 1)

### The crucial role of gut microbiotas

#### Role of the gut microbiotas

Gut microbiotas refers to the diverse community of microorganisms that inhabit the human gastrointestinal tract, including bacteria, fungi, viruses, and other microbes [80]. These microorganisms form a complex symbiotic relationship with the host and have profound effects on human health [81]. Of these, bacteria play a dominant role in the gut microbiota, with their diversity, abundance, and impact on the host being the most significant [82]. They contribute substantially to maintaining gut homeostasis, digestion and metabolism, immune regulation, and the gut-brain axis [83]. In nutritional metabolism, *Bifidobacterium* ferments indigestible dietary polysaccharides, such as dietary fiber, to produce SCFAs [84]. These SCFAs serve as an essential energy source for intestinal epithelial cells and regulate the host’s glucose and lipid metabolism [85]. In pharmacokinetics and pharmacodynamics, *Lactobacillus* within the phylum *Firmicutes* improves intestinal barrier function by secreting SCFAs, such as butyrate, thereby enhancing drug absorption in the gut [86]. *Escherichia coli* produces β-glucuronidase, which hydrolyzes certain conjugated drugs, such as glucuronidated drug metabolites [87]. This reactivation allows the drugs to be reabsorbed in the intestines, forming an “enterohepatic circulation” [88]. Furthermore, *Prevotella* species can alter bile salt composition and metabolism, indirectly affecting the emulsification and absorption of lipophilic drugs, such as certain anticancer agents and vitamin K [89]. In antimicrobial protection and immune modulation, *Bifidobacterium* reduces gut pH by producing lactic acid and acetic acid, thereby inhibiting the growth of pathogens like *Salmonella* and *Escherichia coli* [90]. It also stimulates intestinal dendritic cells (DCs) and macrophages to induce anti-inflammatory cytokines, such as IL-10, reducing inflammatory responses [84]. *Lactobacillus* enhances the intestinal mucosal barrier by stimulating epithelial cells to secrete mucus, preventing pathogen invasion [91]. It also produces lactic acid, hydrogen peroxide, and bacteriocins that directly kill or inhibit pathogens [92]. Additionally, *Lactobacillus* promotes IgA secretion and regulates Th1/Th2 balance, thereby enhancing anti-infective immunity [93]. In maintaining gut barrier integrity and gastrointestinal structure, *Bacteroides thetaiotaomicron* induces the expression of small proline-rich protein 2 A (SPRR2A), essential for maintaining epithelial villous desmosomes [94]. The *Lactobacillus rhamnosus* produces two soluble proteins, p40 and p75, which prevent cytokine-induced epithelial apoptosis through epidermal growth factor receptor (EGFR) and protein kinase C (PKC)-dependent pathways [95]. The endocannabinoid system is another entity involved in maintaining gut barrier function mediated by the gut microbiotas. *Akkermansia muciniphila*, a Gram-negative bacterium, increases endogenous cannabinoid levels, enhancing gut barrier function by reducing metabolic endotoxemia [96].

In addition to bacteria, fungi, and viruses in the gut also play indispensable roles in maintaining microbial ecological balance, immune regulation, and metabolism [97]. Gut fungi, such as *Candida albicans* and *Saccharomyces cerevisiae*, can activate dendritic cells and T cells, enhancing antigen recognition and maintaining immune homeostasis [98]. Moreover, fungi interact with bacteria to form a complex microbial ecological network. For instance, *Candida albicans* may overgrow during gut microbiota dysbiosis, potentially leading to diseases such as inflammatory bowel disease (IBD) [99]. In terms of viruses, there’s not sufficient research currently being done. The gut virome primarily consists of bacteriophages and certain enteric viruses, such as noroviruses. These viruses contribute to the regulation of bacterial communities, immune modulation, and the potential treatment of bacterial infections [100].

Recent studies have revealed that gut microbiotas play a pivotal role not only in regulating digestion and immune functions but also in influencing host metabolism, inflammatory responses, and even mental health [101]. Dysbiosis, or an imbalance in the gut microbiotas, has been closely linked to the development of various diseases, including obesity, type 2 diabetes, IBD, irritable bowel syndrome (IBS), and cardiovascular disorders [102]. Therefore, maintaining a diverse and balanced gut microbiotas is crucial for disease prevention and the promotion of overall health.

#### Gut microbiotas’ alteration in CRC

Humans are exposed to bacteria from birth, and some will gradually form an individual’s specific gut microbiota environment [103, 104]. Throughout millions of years of human evolution, the gut microbiotas have long been an indispensable member of the human body. A healthy gut microbiota environment even benefits human growth and development [105, 106]. Due to the natural proximity, gut microbiotas are particularly closely related to the intestinal tract, establishing a special mutualistic symbiosis between gut microbiotas and the intestinal tract. A healthy gut microbiota environment is extremely important for an infant’s gut development, especially for the enteric nervous system (ENS) [107, 108]. A study revealed that altered composition of the intestinal flora by ampicillin treatment decreased the number of nitrogen neurons in the colon of adult mice, inhibiting neurogenesis and thereby reducing the colon’s motor capacity [109]. Intestinal flora carries out complex and active metabolic activities in the human intestine, utilizing various substances in the host intestine to produce many harmful or beneficial metabolites, such as lipopolysaccharide, peptidoglycan, trimethylamine, secondary bile acids, and short-chain fatty acids [110–114]. It also regulates inflammation and the adaptive and innate immune systems [115].

CRC is the most common malignant tumor of the human colon and rectum. Relevant studies have found that patients with colorectal cancer commonly exhibit dysbiosis in their gut microbiotas, with significant differences in the composition and relative abundance of microbial communities compared to healthy individuals [110]. Pathogenic bacteria such as *Fusobacterium nucleatum*, *Parvimonas micra*, *Peptostreptococcus anaerobius*, and *Solobacterium moorei* are enriched, while beneficial bacteria like *Streptococcus thermophilus* and *Lactobacillus gallinarum* are depleted [116]. These findings suggest that changes in the gut microbiotas play a key role in the initiation and progression of CRC. It is not surprising that there is a close association between the occurrence and development of CRC and gut microbes. In contrast to gastric cancer, where a single microbiota is dominant [117], identifying oncogenic culprits from the colonic microbiotas and determining their participation in the development of CRC is extremely challenging. Changes in the characteristics of the gut microbiotas have been reported to be consistently present in CRC, with tumor characteristics differing from those of adjacent normal tissues. These differences consist of decreased diversity and changes in the community composition, and they increase with the progression of CRC [118–122]. Analyzing the fecal gut microbiotas of CRC patients and controls has identified significantly altered gut microbiotas in CRC [123]. Throughout CRC progression, changes in gut microbiotas are not constant. The majority (60%) of CRC cases arise via adenomas. During the onset of CRC, *Actinomyces*, *Streptococcus, Lactobacillus zeae*, *Dorea*, *Lachnospiraceae*, and *Peptostreptococcus anaerobius* are enriched, while *Clostridia* is decreased in adenomas patients [124, 125]. In CRC progression, marked alterations are observed in *Streptococcus bovis*, *Escherichia coli*, *Fusobacterium nucleatum*, *Salmonella enterica*, *Enterotoxigenic Bacteroides fragilis*, *Enterococcus faecalis* and *Clostridium septicum* [126]. In the metastatic stage of CRC, *Escherichia coli*, *Fusobacterium nucleatum*, and *Bacteroides fragilis* meet more significant changes [126–128]. There is no doubt that these changes in gut microbiotas play an important role in CRC. Macroscopically, gavage of fecal from CRC patients promotes colorectal carcinogenesis in mouse model [129]. The mechanism behind this phenomenon is quite complex and not entirely clear. Partial studies now show that certain intestinal bacteria are directly involved in CRC cell biology behavior or life activities, and almost all of the gut microbiotas mentioned above are involved in the proliferation of CRC cells [85, 86, 130–133].

Studies have also shown that alterations in the gut fungal community may indirectly influence the development of CRC by promoting local immune responses, disrupting intestinal barrier function, or exacerbating inflammatory reactions [134]. The gut fungal community in CRC patients is often characterized by dysbiosis. In healthy individuals, fungi such as *Saccharomyces cerevisiae* are typically present at low levels, whereas in CRC patients, the proportion of certain pathogenic fungi is increased, which may be associated with intestinal inflammation and changes in immune responses [135]. Research indicates that the inflammatory state of the gut may provide a favorable environment for fungal overgrowth. Additionally, the gut virome in CRC patients differs from that of healthy individuals, with certain enteric viruses, such as noroviruses and adenoviruses, being more abundant in CRC patients. The presence of these viruses may be related to immune evasion mechanisms, as they can infect intestinal cells or microbial communities, potentially aiding cancer cells in the tumor microenvironment to evade immune surveillance, thereby accelerating tumor progression.

### The interaction between gut microbiotas and fatty acid metabolism in CRC

#### Gut microbiotas participate in fatty acid metabolism

Gut microbiotas play a crucial role in fatty acid metabolism, influencing the host’s lipid absorption, transformation, and metabolic regulation through multiple mechanisms [136]. These include the production and energy regulation of SCFAs, bile acid metabolism, and fat storage and breakdown, which influence spans across the entire human lifespan.

##### Fetal stage

During fetal development, the gut remains in a nearly sterile state within the maternal womb, and the lack of microbiota means minimal production of SCFAs [137]. Consequently, the fetus primarily relies on maternal fatty acids supplied through placental transport to provide energy and support neural development.

##### Neonatal stage

At birth, the gut is rapidly colonized by microbes, and fatty acids supplied by breastfeeding, such as Omega-3 and Omega-6 fatty acids, are gradually integrated into the lactose-microbiota metabolic system. *Bifidobacterium* species ferment human milk oligosaccharides (HMOs), promoting SCFA production, which aids in the development of the intestinal barrier [138]. Additionally, *Bifidobacterium* supports the production of key microbial metabolites such as propionate, which regulates the secretion of gut hormones like glucagon-like peptide-1 (GLP-1), thereby influencing neonatal energy metabolism and fat storage [139].

##### Infant and childhood stages

With the introduction of complementary foods, the gut microbiota gradually matures and becomes more diverse, resulting in more flexible fatty acid metabolism. Microbial metabolites begin to exert more pronounced effects on gut function. For instance, *Lactobacillus* species enhance SCFA synthesis, particularly butyrate, which plays a crucial role in energy provision, gut barrier regulation, and immune modulation [140].

##### Adulthood

As individuals age, the gut microbiota stabilizes, and the gut barrier function strengthens. However, lifestyle factors such as diet and antibiotic use can significantly impact the microbial ecosystem. Dietary patterns, such as the high-fat Western diet, can disrupt the microbial balance, leading to an increased proportion of pro-inflammatory strains like *Escherichia coli* and *Firmicutes*, which influence fatty acid oxidation and storage [141]. This imbalance can result in gut dysbiosis and lipid metabolic disorders.

##### Elderly stage

In the elderly, gut microbiota diversity tends to decline, with a notable reduction in beneficial strains like *Lactobacillus*, which can negatively impact lipid metabolic homeostasis and contribute to metabolic disorders such as type 2 diabetes [142]. Therefore, supplementing the diet with prebiotics and probiotics can help restore fatty acid metabolic balance and promote gut health in older adults.

It can be seen that SCFAs are the ones in fatty acid metabolism where changes in the gut microbiotas have a more pronounced effect on the organism. SCFAs are known as significant microbial metabolites [143]. *Clostridium butyricum*, *Bifidobacterium, Lactobacillus rhamnosus, Streptococcus thermophilus, Lactobacillus reuteri, Lactobacillus casei*, and *Lactobacillus acidophilus* are the most prevalent microbes that produce SCFA [144]. *Clostridium butyricum* inhibits the Wnt/β-catenin signaling cascade by reducing HDAC activities, limiting the proliferation of intestinal cancers in a mouse model [133]. *Lactobacillus rhamnosus* reduces CRC burdens and boosts anti-tumor immunity in mouse models in a CD8 + T cell-dependent manner [145]. When accompanied by CTLA-4-blocking antibodies, *Lactobacillus reuteri* dramatically reduced the population of antibiotic-resistant bacteria and organized a tumor-fighting immunity in mouse CRC model [146–148]. The *Lactobacillus casei* bacteria orally administered to mice that have tumors can inhibit the development of tumors by triggering immune system reactions [149]. Murine models show that *Streptococcus thermophilus* suppresses tumors by secreting β-galactosidase, which activates oxidative phosphorylation, which inhibits Hippo process kinase and increases the quantity of *Bifidobacterium* and *Lactobacillus* [150]. *Bifidobacterium* hinders the formation of cancer cells in CRC by halting the cell cycle in the G0/G1 phase and raising the activity of alkaline phosphatase, a unique marker that is reduced in malignant cells [144]. *Faecalibaculum rodentium* and its human homolog *Holdemanella biformis* are two intestinal strains with antitumor effects that protect the gut from tumor growth primarily through the production of SCFAs [151, 152]. These SCFAs prevent the activation of calmodulin phosphatase and nuclear factor of activated T-cells C3 (NFATc3) by inhibiting histone deacetylases (HDACs), which works in both mice and human [151]. Another SCFA-producing bacterium and potential protective probiotic, *Akkermansia muciniphila*, promotes the enrichment of M1-type macrophages in mice, thereby inhibiting CRC development [153]. (Table 1).

**Table 1:** Gut microbiotas affecting CRC.

Names	The type of model	Mechanism	References
*Akkermansia muciniphila*	Human	Suppress CRC by increasing the number of butyrate-forming probiotics	[9]
*Clostridium butyricum*	Murine	Limit the pathway mediated by Wnt/β-catenin via suppressing HDAC activity	[127]
*Lactobacillus rhamnosus*	Murine	Decrease CRC incidence and boost anti-tumor immune defense in a CD8 + T cell-dependent manner	[135]
*Lactobacillus reuteri*	Murine	Reduce the population of antibiotic-resistant bacteria by combining the CTLA-4 blocking antibodies	[136–138]
*Lactobacillus casei*	Murine	Through activating immune systems in tumor-bearing mice	[139]
*Streptococcus thermophilus*	Murine	Secret β-galactosidase by activating oxidative and inhibiting Hippo process kinase	[140]
*Bifidobacterium*	Murine	Halt the cell cycle at the G0/G1 phase and increase alkaline phosphatase activity	[134]
*Faecalibaculum rodentium* and *Holdemanella biformis*	Murine and human	Suppress CRC development via suppressing HDACs and preventing calcineurin and NFATc3 activation	[141, 142]
*Akkermansia muciniphila*	Murine and human	Boost the proliferation of M1-like macrophages in mice to suppress the progression of CRC	[143]

*CRC* colorectal cancer, *HDAC* histone deacetylase, *CTLA-4* cytotoxic T lymphocyte-associated antigen-4, *SCFAs* short-chain fatty acids, *NFATc3* nuclear factor of activated T cells C3.

Metabolites produced from the gut microbiotas were found to have a significant effect on immunotherapy effectiveness for particular cancer types. The disturbance of gut microbiotas can lead to the decrease of SCFAs production, and the decrease of SCFAs can further aggravate the disturbance of gut microbiotas and even lead to the occurrence and development of CRC. Therefore, appropriate supplementation of SCFAs along with probiotics has the potential to break this vicious cycle and thus inhibit the further deterioration of CRC.

#### Metabolic reprogramming of fatty acid induces changes in gut microbiotas

In recent years, with the deepening research on gut microbiotas, it has become evident that diet can significantly influence the structure and function of gut microbiotas. For example, HFDs can increase the abundance of *Firmicutes* while reducing beneficial bacteria such as *Bacteroides* and *Lactobacillus* [107, 108]. Dietary interventions not only reshape the gut microbiota composition but also affect microbial metabolism [105, 106]. Among these, fatty acid metabolites act as signaling molecules mediating the material-energy metabolism and immune regulation between gut microbes and their host. The dynamic balance between gut microbiotas and host fatty acid metabolism is crucial for maintaining host health [154]. In CRC patients, an apparent dysregulation of gut metabolism is observed, particularly in fatty acid metabolism. This metabolic disruption significantly alters the gut microbiota composition, making fatty acid metabolism one of the most direct and critical factors influencing gut microbial dynamics [155].

Studies suggest that CRC patients may accumulate more SFAs such as palmitic acid and stearic acid, possibly due to increased demand by tumor cells to support their growth and proliferation [146]. This could also result from the upregulation of enzymes like FASN and acetyl-CoA carboxylase (ACC), which convert acetyl-CoA into SFAs [147, 148]. The accumulation of SFAs contributes to gut microbial dysbiosis, promoting the proliferation of sulfate-reducing bacteria such as *Bilophila wadsworthia* and inflammatory bacteria like *Enterococcus faecalis*, while suppressing beneficial microbes. This microbial imbalance has been shown to induce colitis in genetically predisposed IL-10-deficient mice [146]. In another study, high-SFA intake in C57BL/6 J mice led to an increased abundance of three sulfate-reducing bacteria in the colonic mucosa [156]. This also reduced the expression of tight junction protein genes, increasing intestinal permeability and lipopolysaccharide-binding protein levels. Metabolic byproducts such as H₂S gas, produced by these bacteria, disrupt the intestinal barrier and trigger endotoxemia [157, 158]. Overall, alterations in fatty acid metabolism significantly modify gut microbiota composition, disrupt intestinal homeostasis, and promote IBD and CRC development.

Unsaturated fatty acids (UFAs), particularly polyunsaturated fatty acids (PUFAs) such as Omega-3 and Omega-6 fatty acids, play a crucial role in regulating gut microbiota metabolism [159]. They influence gut microbial composition, functionality, and metabolic activity through direct and indirect pathways. Omega-3 fatty acids, including α-linolenic acid, EPA, and DHA, promote the growth of beneficial bacteria like *Lactobacillus* and *Bifidobacterium* [160]. By increasing the abundance of these probiotics, Omega-3 fatty acids help reduce pro-inflammatory cytokines such as IL-6 and TNF-α, regulate immune responses, and mitigate chronic gut inflammation, creating a favorable environment for beneficial microbes [161]. The effects of Omega-6 fatty acids remain controversial. While moderate intake of Omega-6 fatty acids offers health benefits, excessive consumption can induce inflammatory responses, indirectly impacting gut microbiotas [30]. High doses of Omega-6 fatty acids, particularly linoleic acid, may exacerbate gut inflammation by promoting the release of pro-inflammatory cytokines, altering gut microbiota composition, and fostering the proliferation of pathogenic bacteria [162]. Research shows that excessive Omega-6 fatty acids intake can increase the abundance of pathogenic species like *Fusobacterium nucleatum* while reducing beneficial microbes [163]. For instance, overconsumption of Omega-6 fatty acids in C57BL/6 J mice increased *Proteobacteria* populations and macrophage infiltration in adipose tissue [164]. Conversely, supplementation with Omega-3 fatty acids derived from fish oil reduced the abundance of inflammation-associated gut bacteria such as *Enterobacteriaceae* and *Clostridium* cluster in C57BL/6 J mice while increasing beneficial bacteria such as *Akkermansia*, *Lactobacillus*, and *Bifidobacterium* [164]. These beneficial microbes enhance gut health by recruiting regulatory T cells to the small intestine, suppressing bacterial overgrowth, and mitigating inflammation [165]. As a result, a balanced ratio of Omega-6 to Omega-3 fatty acids in the diet is critical for maintaining gut microbial health [33]. Increasing Omega-3 fatty acids intake while limiting excessive Omega-6 fatty acids consumption not only improves gut microbiota composition but also promotes the growth of beneficial bacteria [166]. Furthermore, these dietary adjustments can reduce fatty acid metabolism dysregulation, enhance intestinal barrier integrity, and positively impact overall gut health.

**Fig. 2:**
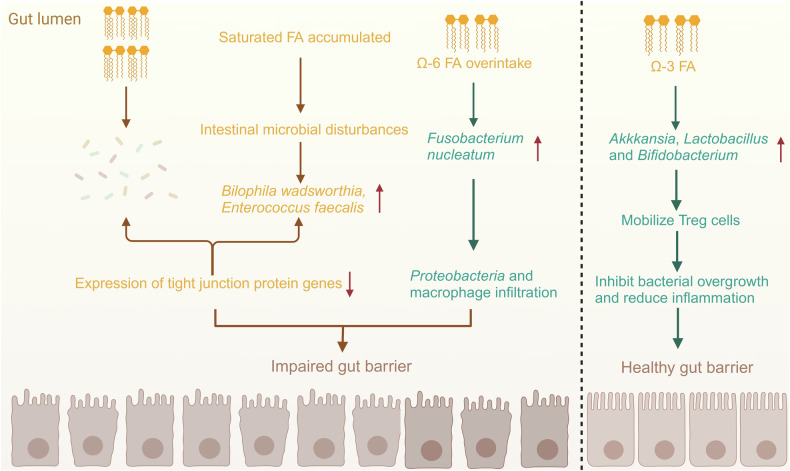
Metabolic reprogramming of fatty acid induces changes in gut microbiotas.

Conclusively, as one of the most important nutrients in the human body, the metabolic pattern of fatty acids is the most important factor in shaping intestinal microflora [167]. The type and quantity of fatty acids will affect the composition and quantity of intestinal microflora. At the same time, intestinal microorganisms can participate in the metabolic regulation of the host, thus affecting the intestinal barrier function, and thus affecting the health of the host [168] (Fig. 2).

#### Metabolic reprogramming of fatty acid and gut microbiotas’ alteration may form positive feedback in CRC

The gut microbiota serves as a “bridge” between fatty acids and the host, influencing host fatty acid metabolism through a microbe-host co-metabolism network [169, 170], which in turn affects fatty acid digestion and absorption. Conversely, fatty acid over-intake also impacts the gut micro-ecosystem, altering the structure of gut microbiota and regulating its metabolites [171]. Some of these metabolites can cross the intestinal barrier and enter the bloodstream, where they modulate various signaling pathways and metabolic cycles in the host, thereby influencing overall metabolism and health [172, 173]. Among these, HFDs have the most significant impact. HFDs promote gut microbiota dysbiosis, leading to a noticeable shift in the *Firmicutes/Bacteroidetes* ratio, which in turn results in a reduced production of SCFAs [174]. Furthermore, the excessive intake of SFAs in HFDs exerts pro-inflammatory effects, potentially inducing lipid peroxidation and generating carcinogenic metabolites such as malondialdehyde (MDA) and 4-hydroxy-2-nonenal (4-HNE) [175].

In CRC patients, significant reductions in both SCFA levels and probiotics are observed, highlighting the interplay between these factors in CRC development and progression. As the primary energy source for colonic epithelial cells, SCFAs can modulate broad immune responses and act as tumor suppressors by regulating gene expression through epigenetic mechanisms [176]. Moreover, SCFAs have significant roles in the prevention of intestinal diseases and exhibit anticancer properties [177]. They are essential for maintaining T-cell homeostasis and exert effects such as anti-inflammatory and immunomodulatory actions, downregulation of the Wnt/β-catenin signaling pathway associated with CRC, restriction of tumor cell growth and migration, inhibition of tumor development, induction of apoptosis, and promotion of CRC cell differentiation [178–180]. SCFAs mainly include acetate, propionate, and butyrate, which are produced in the gut in different proportions: acetate (60%), propionate (25%), and butyrate (15%) [181, 182]. The optimal ratio of these SCFAs is 3:1:1, respectively, with butyrate being the most extensively studied SCFA [183]. Butyrate is primarily produced through glycolysis by hydrocarbon-degrading bacteria, such as *Faecalibacterium prausnitzii* from the *Ruminococcaceae* and *Lachnospiraceae* families [179]. It plays a pivotal role in influencing immune system function, maintaining intestinal barrier integrity, enhancing the efficacy of anticancer therapies, and reducing the incidence of chemotherapy-induced mucositis [184]. In healthy colonic cells, butyrate undergoes mitochondrial β-oxidation, providing energy through the tricarboxylic acid (TCA) cycle or cytosolic acetyl-CoA [185]. However, in CRC cells, butyrate uptake is reduced due to metabolic reprogramming, such as the Warburg effect, wherein cancerous cells prefer glucose metabolism over fatty acid oxidation. Furthermore, butyrate can inhibit CRC progression by disrupting the AKT/ERK signaling pathway and reducing CRC cell motility in an HDAC3-dependent manner. It also activates transcription of apoptosis-related genes like p21/Cip1, leading to G1-phase cell cycle arrest [186, 187]. In CRC patients, the abundance of butyrate-producing bacteria such as *Faecalibacterium prausnitzii* and *Roseburia spp*. is significantly reduced, contributing to a marked decline in butyrate levels [188, 189]. This decrease is further exacerbated by metabolic reprogramming in CRC cells, reducing butyrate utilization. In mouse models and in vitro experiments, butyrate was shown to inhibit SIRT3, triggering a pyruvate dehydrogenase complex to channel glycolytic byproducts into the TCA cycle, thereby altering the Warburg effect and promoting cancer cell death [190].

Moreover, studies have shown that the interaction between gut microbiota and fatty acid metabolism plays a crucial regulatory role in the development and progression of CRC through the gut-brain axis [191]. Fatty acid metabolites produced by gut microbiota, such as SCFAs, can cross the blood-brain barrier and influence the hypothalamic-pituitary-adrenal (HPA) axis, stimulating the secretion of gut hormones such as glucagon-like peptide-1 (GLP-1) and peptide tyrosine tyrosine (PYY), which in turn regulate lipid metabolism and inflammatory responses [192, 193]. Among them, acetate can directly cross the blood-brain barrier and act on the arcuate nucleus (ARC) of the hypothalamus, activating dietary nutrient-sensing signals such as GPR43/41 receptors to regulate hypothalamic responses to food intake, reduce appetite, and decrease lipid synthesis in the liver and adipose tissue [194]. Chronic stress can influence fat storage through sympathetic nervous system activation, thereby altering the CRC microenvironment and affecting the energy supply to cancer cells [195]. Consequently, dysbiosis of the gut microbiota and disruptions in fatty acid metabolism can indirectly promote CRC progression by influencing stress responses and neuroinflammation.

**Fig. 3:**
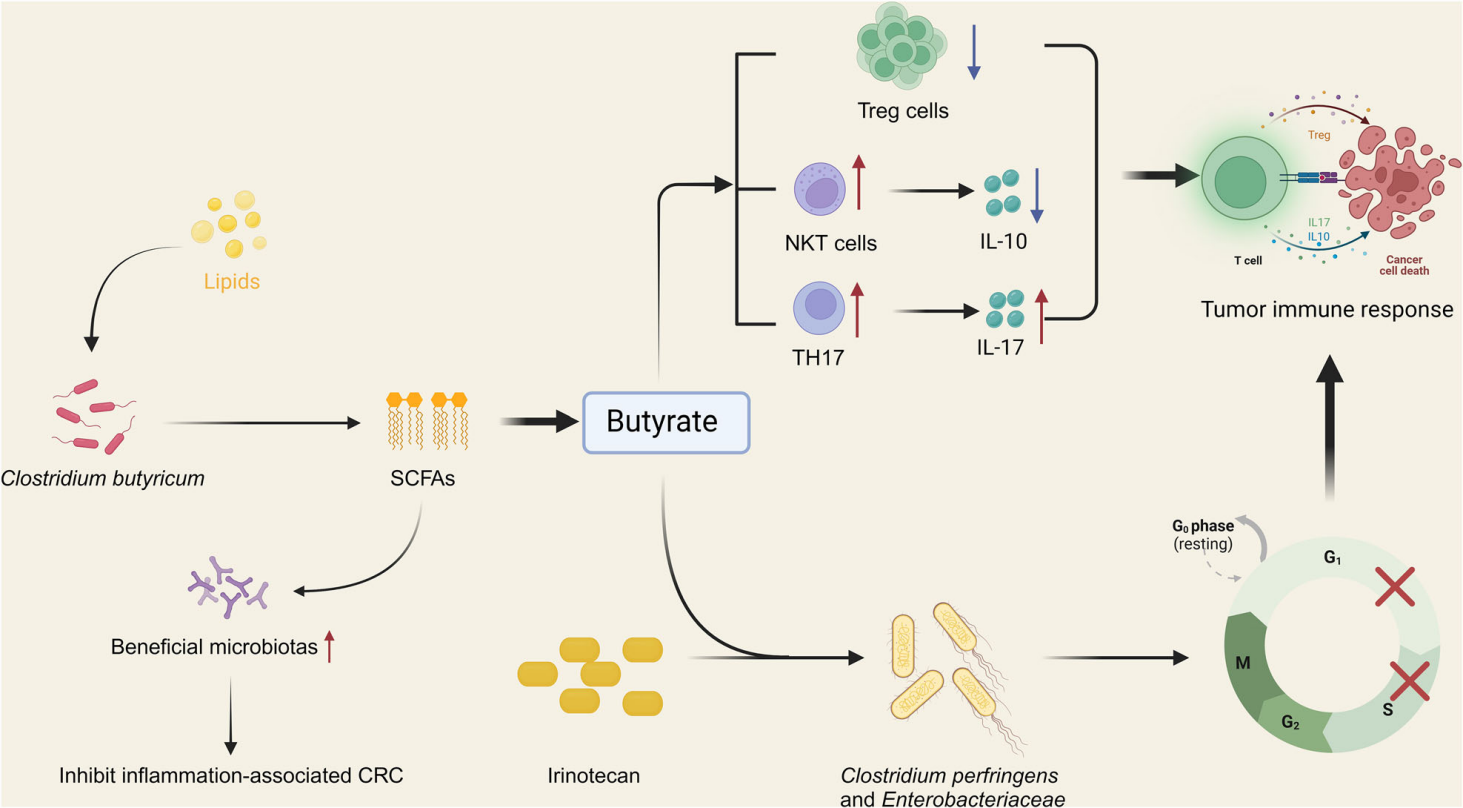
Metabolic reprogramming of fatty acid and gut microbiotas’ alteration may form positive feedback in CRC.

These changes in the gut microenvironment create a positive feedback loop in CRC patients. Alterations in the gut microbiota and remodeling of fatty acid metabolism sustain the growth of CRC tumor cells [184]. The reduction in butyrate levels due to the decreased abundance of *Faecalibacterium prausnitzii* not only impairs immune system function but also compromises intestinal barrier integrity, further decreasing butyrate-producing bacteria [196]. In turn, the reduction of these bacteria leads to lower SCFA levels, weakening immunoregulatory and anti-inflammatory mechanisms and contributing to inflammation-associated CRC development. (Fig. 3).

### Future directions and opportunities

Currently, there is growing interest in understanding the impact of gut microbiota on cancer therapies. This influence is often bidirectional, as many drugs are regulated by the gut microbiota, leading to individual variability in drug metabolism, which in turn significantly affects the efficacy and side effects of drugs for various disease indications [197]. Conversely, systemic therapies also alter the composition and function of the microbiota. Fatty acid metabolism and gut microbiota play crucial roles in the development and progression of CRC [198]. Therefore, exploring the interplay between fatty acid metabolism and gut microbiota holds promise for providing novel therapeutic opportunities for CRC diagnosis and treatment.

Future research should focus on elucidating the dynamic interactions between gut microbiota and fatty acid metabolism and how these interactions jointly influence host health. For instance, precision regulation of microbial metabolites could be investigated, using multi-omics approaches such as metabolomics and microbiomics to analyze how specific microbial populations regulate the production and utilization of metabolites like SCFAs [199]. Studies could also explore how fatty acid intake reshapes the structure and metabolic potential of gut microbiota and how long-term host-microbiota metabolic interactions alter metabolic homeostasis and disease risk [200]. Additionally, leveraging the microbiota’s capacity to regulate fatty acid metabolism could lead to more effective interventions. For example, the development of targeted probiotics or probiotic consortia, such as butyrate-producing strains, could optimize the gut microbiota to enhance SCFA production and utilization [201]. Investigating the microbiota-metabolic networks in different populations may facilitate personalized dietary interventions based on individual microbiome profiles. Furthermore, new drugs or biomaterials targeting microbial metabolites or associated signaling pathways could be developed to promote healthy fatty acid metabolism and improve treatment outcomes for metabolic-related diseases [202]. In the realm of CRC diagnosis, biomarkers based on fatty acid metabolism or microbiota-derived metabolites could be developed for early screening and diagnosis, enabling timely intervention in the early stages of disease progression.

The integration of emerging technologies into the study of fatty acid metabolism and gut microbiota represents another exciting frontier. Multi-omics approaches, including high-resolution mass spectrometry and nuclear magnetic resonance (NMR), allow precise detection of SCFAs and other fatty acid metabolites, facilitating the analysis of the relationship between gut microbiota metabolic networks and host metabolism [203]. Genomics and transcriptomics can analyze the functional genes and expression profiles of gut microbiota, uncovering microbial metabolic potential and its connection to fatty acid metabolism [199]. Single-cell sequencing and spatial transcriptomics enable the study of gut microbiota-host cell interactions, identifying the contributions of different cell types to fatty acid metabolism and visualizing the spatial distribution of fatty acid metabolites in the gut microenvironment [204]. These approaches help elucidate the localized functions of specific metabolites and microbial populations.

Overall, future research should leverage interdisciplinary collaboration and innovative technologies to systematically analyze the relationship between gut microbiota and fatty acid metabolism, ultimately facilitating the comprehensive translation of basic research into clinical applications. This advancement will deepen our understanding of fatty acid metabolism and gut microbiota, providing new possibilities for the prevention, diagnosis, and personalized treatment of diseases.

## Conclusion

Accumulating evidence suggests that the tumor microenvironment largely supports cancer progression by promoting cancer cell growth, remodeling metabolism, and suppressing the immune response. We live in a world that is increasingly concerned with microbiotas, and although our understanding is still in its infancy, we do know that changes in the gut microbes to the gut microenvironment are critical to the development of CRC. Similarly, lipid metabolism occurs differently in tumor cell metabolism. Fatty acid metabolic pathways in CRC patients may be potential specific targets for CRC therapy. SCFAs derived from gut microbes can potentially have a significant impact on the diagnosis and prognosis of CRC. Further work should focus, as a priority, on managing the microbiome and metabolome to modulate the gut micro-ecology to activate a potent anti-cancer effect. This might offer promising and desirable applications for CRC therapy regarding sustainability in terms of safety, utility, and affordability. The association between gut microbiotas and fatty acid metabolism and the efficacy of anticancer therapies may be a potential new approach to the treatment of CRC. Bacteria that produce SCFAs may improve the chemotherapeutic agents’ pharmacological effects and the host’s response to immunotherapies, thus strengthening the therapeutic response to CRC. Therefore, combining gut microbiotas with fatty acid metabolism will certainly be a promising direction. In addition, future studies could explore more about the relationship between fungi and fatty acid metabolism and colorectal cancer by studying gut microbiota, expanding beyond the scope of gut bacteria.
